# Persistent emotional stress, fatigue and impaired neurocognitive function in recovered COVID-19 patients: a longitudinal prospective study

**DOI:** 10.1192/j.eurpsy.2022.1261

**Published:** 2022-09-01

**Authors:** A. Rogiers, S. Launay, G. Duque, E. Soukias, S. Van Eycken, T. Besse-Hammer, D. Sanchez-Rodriguez, M. Chalon, M.-D. Gazagne, E. Maillart, F. Benoit, M. Surquin, F. Corrazza, O. Michel, C. Kornreich

**Affiliations:** 1 CHU Brugmann, Department Of Psychiatry, Brussels, Belgium; 2 Vrije Universiteit Brussel, Faculty Of Medicine, Brussels, Belgium; 3 CHU Brugmann, Clinical Trial Department, Brussels, Belgium; 4 CHU Brugmann, Geriatry, Brussels, Belgium; 5 CHU Brugmann, Physical Medicine And Rehabilitation, Brussels, Belgium; 6 CHU Brugmann, Neurology, Brussels, Belgium; 7 CHU Brugmann, Department Of Infectious Diseases, Brussels, Belgium; 8 CHU Brugmann, Immunology, Brussels, Belgium; 9 Université Libre de Bruxelles, Faculty Of Medicine, Brussels, Belgium

**Keywords:** Covid-19, cognitive function, post-traumatic stress symptoms, Anxiety

## Abstract

**Introduction:**

Several surveys report that post-COVID-19 patients (pts) could be at risk of persistent emotional distress, fatigue and impaired neurocognitive function (NCF).

**Objectives:**

The aim was to assess emotional distress, fatigue and NCF in order to provide adequate care.

**Methods:**

Patients with persistent physical or mental symptoms, at least 8 weeks post-COVID-19, were eligible for this ongoing prospective longitudinal single center trial. Data on depression, anxiety, cognition, post-traumatic stress symptoms (PTSS) and fatigue were collected using 4 validated questionnaires at study entry (T0) and at 6 months (T1).

**Results:**

Ninety-three pts were recruited between November 2020-March 2021. Test results from 64 eligible pts (15 male pts) were analyzed at T0; 63 pts (98%) were treated in outpatient settings. Median age was 47 years [range 27-75]). Median time since COVID-19 was 29 weeks [range 8-53]. Twenty-two pts (34%) had a history of psychiatric disorders. According to the Hospital Anxiety Depression Scale (HADS), 44 pts (73%) reported anxiety symptoms and 26 pts (41%) reported depressive symptoms; 48 pts (69%) reported cognitive complaints according to the Cognitive Failure Questionnaire and 29 pts (45%) suffered from PTSS, according to the Post-Traumatic Stress Disorder Checklist-Civilian Version (PCL-C). Fifty-five pts (86%) had an elevated score on the Fatigue Severity Scale, indicating severe fatigue. Twenty-seven pts (42%) were still on sick leaf. Diminished social support and psychiatric history were predictive factors for neurocognitive dysfunction and PTSS.

**Conclusions:**

A majority of patients who recovered physically from COVID-19, are at risk for suffering from persistent anxiety, PTSS and neurocognitive dysfunction.

**Disclosure:**

No significant relationships.

